# Incidental finding of MELAS in a young woman with decompensated heart failure and end stage kidney disease: a case report

**DOI:** 10.1093/ehjcr/ytae690

**Published:** 2024-12-24

**Authors:** Novi Yanti Sari, Ching-Hui Sia, Raymond Ching Chiew Wong, Weiqin Lin, Yoke Ching Lim

**Affiliations:** Department of Cardiology, National University Heart Center Singapore, 5 Lower Kent Ridge Rd, Singapore, Singapore 119074; Department of Cardiology, National University Heart Center Singapore, 5 Lower Kent Ridge Rd, Singapore, Singapore 119074; Department of Cardiology, National University Heart Center Singapore, 5 Lower Kent Ridge Rd, Singapore, Singapore 119074; Department of Cardiology, National University Heart Center Singapore, 5 Lower Kent Ridge Rd, Singapore, Singapore 119074; Department of Cardiology, National University Heart Center Singapore, 5 Lower Kent Ridge Rd, Singapore, Singapore 119074

**Keywords:** Case report, Heart failure, Kidney disease, MELAS

## Abstract

**Background:**

Mitochondrial encephalopathy, lactic acidosis, and stroke-like episodes (MELAS) is a rare and progressive mitochondrial disorder characterized by multi-systemic involvement. This disease manifests in various clinical manifestations, with heart and kidney disorders being among the most common. Accurate diagnosis of MELAS often necessitates a range of complex investigations. Prompt and comprehensive management can significantly improve the prognosis of the disease.

**Case summary:**

A 40-year-old female presented with elevated blood pressure (BP) associated with shortness of breath prior to dialysis. She was found to be hypertensive with a systolic BP of 190 mmHg with prominent signs of congestion. Laboratory examination showed elevated troponin and NT-proBNP. Arterial blood gas revealed severe lactic acidosis, which prompted urgent dialysis. On the latest admission, an echocardiogram showed a left ventricular ejection fraction of 50% with much thickened myocardium compared with the previous study. Linking the past history of hearing impairment, kidney disease, giddiness, and progression of myocardial thickness warranted a genetic test, which revealed the diagnosis of MELAS.

**Discussion:**

This case involved a patient initially diagnosed with hypertensive heart disease based on asymptomatic left ventricular hypertrophy. Further deterioration led to the identification of MELAS syndrome through extensive diagnostic evaluation. This highlights the importance of considering mitochondrial diseases in unexplained cardiac symptoms, especially in younger patients, for timely and appropriate management.

**Learning points**

- In young patients with unexplained cardiac symptoms, particularly with hypertrophic changes and renal or neurological history, mitochondrial disorders like MELAS should be considered.
- Genetic testing is key in confirming mitochondrial disease, enabling tailored treatment, and family counselling.
- MELAS treatment demands a multi-disciplinary approach, including therapies that reduce mitochondrial dysfunction and avoid mitochondrial-toxic drugs.

## Introduction

Mitochondrial encephalopathy, lactic acidosis, and stroke-like episodes (MELAS) is a rare, progressive mitochondrial disorder caused by maternally inherited mutations in mitochondrial DNA (mtDNA) characterized by diverse clinical manifestations that develop gradually, including strokes, headaches, seizures, cognitive decline, hearing loss, and muscle weakness.^1,2^ While MELAS primarily affects the nervous system, it can also involve the cardiovascular and renal systems, leading to cardiomyopathy and heart failure (HF) due to inadequate adenosine triphosphate (ATP) production in about one-third of cases.^3,4^ Chronic kidney disease (CKD), a significant cause of morbidity and mortality in MELAS, necessitates considering mitochondrial disorders in patients with atypical features or relevant family history.^5,6^

## Summary figure

**Figure:**
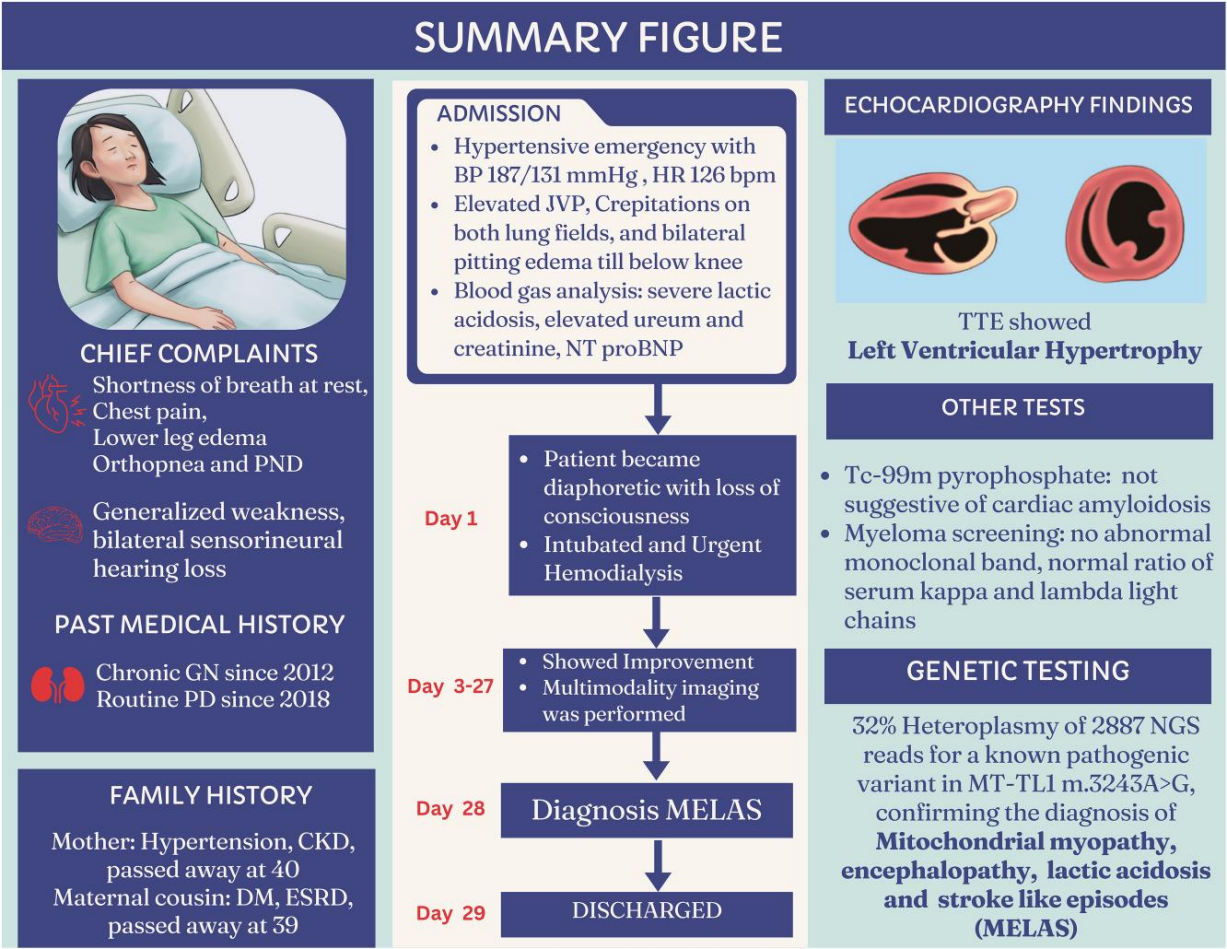


Given the broad clinical spectrum of MELAS and its potential multi-organ involvement, a multi-disciplinary approach is crucial for accurate diagnosis and comprehensive management. Timely recognition can facilitate appropriate interventions and potential treatment strategies aimed at addressing mitochondrial dysfunction, thereby potentially improving patient outcomes and quality of life. In this report, we present a case of incidental findings of MELAS in a patient with decompensated HF and end stage CKD.

## Case summary

A 40-year-old female was referred to our emergency department with elevated blood pressure (BP) of 190/110 mmHg, shortness of breath, chest pain, orthopnea, dizziness, and generalized weakness. Physical examination revealed elevated jugular venous pressure, decreased breath sounds, and lower limb oedema. She was underweight with a BMI of 16.8 kg/m^2^.

She had a history of CKD secondary to chronic glomerulonephritis since 2012 and has been undergoing dialysis since 2018. In 2015, she was referred to cardiology with an echocardiogram indicating hypertensive heart disease and dipyridamole myocardial perfusion imaging showing no signs of ischaemia. No further investigations were pursued. Other past medical history includes young-onset hypertension with primary hyperaldosteronism, recurrent vertiginous dizziness, headaches, and bilateral profound sensorineural hearing loss of unknown aetiology, requiring a right cochlear implant in 2019. An MRI of the internal auditory meatus and brain showed no abnormal mass or significant signal changes, aside from non-specific sub-cortical, periventricular, and deep white matter FLAIR hyperintensities. She also experienced recurrent hospital readmissions due to hypertensive urgency and fluid overload, necessitating dialysis. There was no known history of developmental delay.

Her family history includes her mother with hypertension and CKD, but who subsequently passed away at the age of 40, and her maternal cousin with diabetes mellitus complicated by CKD, who passed away at 39. Her medication included losartan 25 mg once daily (OD), spironolactone 25 mg OD, furosemide 80 mg twice a day (BID), atorvastatin 20 mg OD, ferric polymaltose 100 mg OD, and erythropoietin injection 4000 units three times a week.

**Figure 1:**
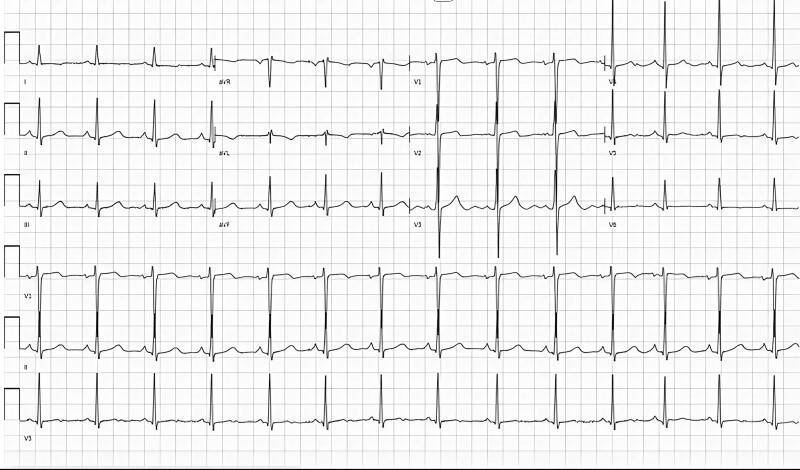
Electrocardiogram demonstrated normal sinus rhythm.

**Figure 2:**
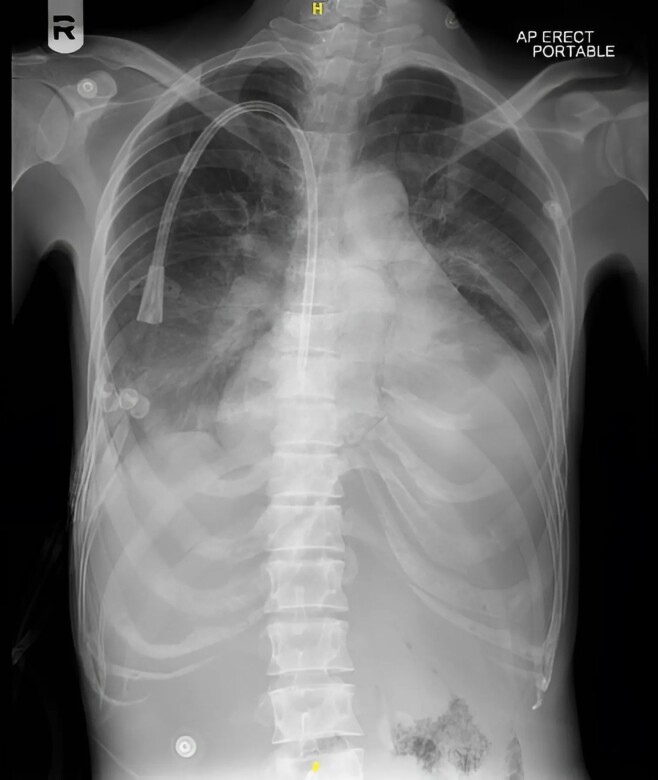
Chest X-ray revealed cardiomegaly, increased pulmonary vascular markings, mild pleural effusion, and a dialysis catheter *in situ*.

Early examinations included electrocardiogram (ECG) (*Figure 1*) and chest X-ray (CXR) (*Figure 2*). Her laboratory result showed haemoglobin at 7.4 g/dL (reference range: 11.6–15 g/dL), troponin at 816.5 ng/L (reference range: <14 ng/L), NT-proBNP >35.000 pg/mL (reference range: 125 ng/L), and creatinine at 388 μmol/L (reference range: 52.2–91.9 μmol/L). She was non-diabetic, and her thyroid function was within normal limits.

Due to elevated troponin and chest pain, she was referred to cardiology for possible coronary angiography. Intravenous nitroglycerin was initiated for high BP. However, she suddenly complained of lightheadedness with diaphoresis, and her BP increased to 236/192 mmHg. Blood gas analysis showed severe lactic acidosis with pH 6.95 and lactate 20 mmol/L (reference range: 0.5–2.2 mmol/L). She was intubated and transferred to the ICU for dialysis.

**Figure 3:**
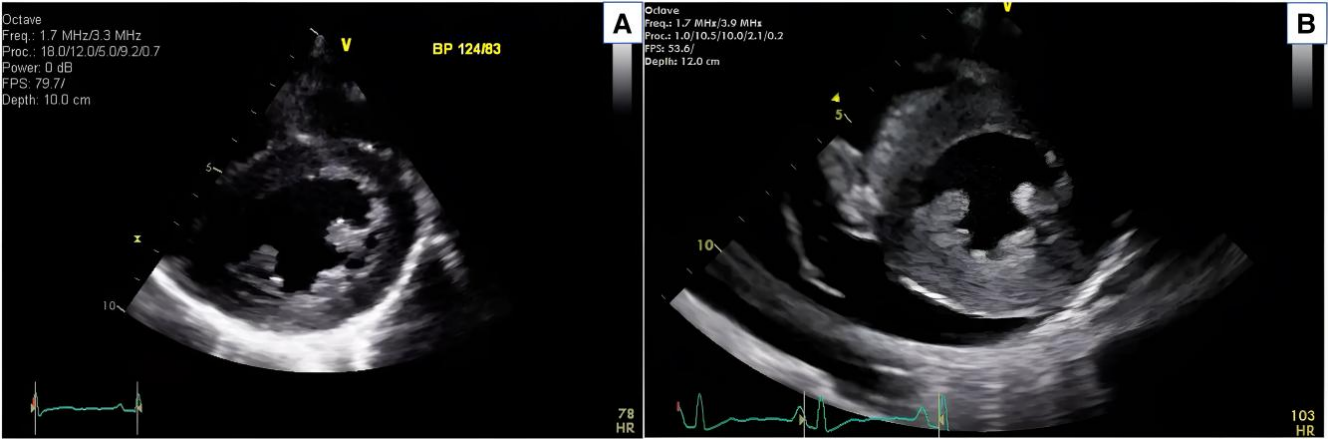
Echocardiogram showed *(A)* initial transthoracic echocardiogram (TTE), *(B)* follow-up TTE.

A transthoracic echocardiogram (TTE) (*Figure 3*) showed a marked increase in left ventricular maximal wall thickness, from 11 mm 6 years earlier to 21.3 mm. The left ventricular ejection fraction dropped from 57% to 50%. There was moderate pericardial effusion without signs of tamponade, along with moderate right ventricular systolic dysfunction and a restrictive filling pattern in the left ventricle. No cardiac mass was seen.

At the ICU, she was started on dialysis using lactate-free dialysate to reach a target dry weight of 32 kg. She received epoetin beta, amlodipine 7.5 mg and aspirin 80 mg OD. Her prior medications were temporarily stopped. She improved significantly after dialysis and was extubated by the third day of her ICU stay.

Further investigations, including Tc-99m pyrophosphate, did not suggestive of cardiac amyloidosis. Myeloma screening revealed no abnormal monoclonal band, and the ratio of serum kappa and lambda light chains was normal (1.18; reference range, 0.26–1.65). Given her strong maternal family history, she underwent genetic testing, which revealed 32% heteroplasmy of 2887 NGS reads for a known pathogenic variant in MT-TL1 m.3243A>G, confirming the diagnosis of MELAS. She was discharged with losartan 25 mg OD, furosemide 80 mg BID, levocarnitine 1000 mg three times a day (TID), ubiquinone 200 mg OD, riboflavin 100 mg OD, and arginine 2500 mg TID a day. She was counselled to avoid certain medications in view of mitochondrial cytopathy, such as aminoglycoside antibiotics, HMG-CoA reductase inhibitors, ringer lactate solution, metformin, serotonin receptor agonists, theophylline, and valproic acid.

Following this admission, she had multiple rehospitalizations for hypervolaemia, hyperkalaemia, and lactic acidosis. Losartan was switched to isosorbide mononitrate 30 mg OD, hydralazine 25 mg BID, nifedipine 60 mg OD, and furosemide 120 mg BID. She is currently stable with NYHA class II, requiring haemodialysis five times a week and regular follow-ups at the cardiomyopathy clinic.

## Discussion

MELAS is a maternally inherited mitochondrial disease that typically presents with neurological symptoms such as seizures, stroke-like episodes, and muscle weakness.^1^ Cardiac involvement occurs in up to 30% of patients, leading to cardiomyopathy.^3,7,8^ LVH is one of the most common manifestations and may progress to dilated cardiomyopathy.^9^

The pathophysiology of HF in MELAS is intricately linked to mitochondrial dysfunction in cardiomyocytes. Mitochondria generate ATP through oxidative phosphorylation, which is crucial for normal cellular functions, including cardiomyocyte contractility.^10^ In MELAS patients, mtDNA mutation disrupts the ATP synthesis, leading to decreased ATP and inefficient energy utilization in cardiac muscle cells.^3,10^ Additionally, dysfunctional mitochondria generate excess reactive oxygen species, further damaging mtDNA and proteins by reducing nitric oxide production, perpetuating the cycle of dysfunction.^2,10^ The heart’s high energy demands make it particularly vulnerable to this damage.^3^ Consequently, compromised energy supply and oxidative damage in cardiomyocytes lead to systolic and diastolic dysfunction, eventually resulting in HF.^10,11^

As MELAS predominantly manifests as LVH, it is crucial to consider various conditions that may present as hypertrophic cardiomyopathy (HCM), such as Fabry, Danon, and infiltrative cardiomyopathy. Diagnosis could be challenging, requiring multi-modality imaging with possible genetic testing to differentiate HCM and its phenocopies.^12^

Cardiac involvement in MELAS highlights the need to consider mitochondrial diseases in the differential diagnosis of HF, especially in young patients with unexplained cardiac symptoms. Early recognition and diagnosis are vital for proper management. The treatment is primarily symptomatic and requires a multi-disciplinary approach due to its multi-organ impact.^2^ Supplementation with antioxidants, cofactors, and compounds like L-arginine, coenzyme Q10, creatine, and citrulline has shown benefits in limited clinical trials.^13,14^ Certain medications that negatively affect mitochondrial function should be avoided.^2,14^ The use of HF medications was limited in our case due to kidney failure and recurrent lactic acidosis.

## Conclusion

MELAS, a maternally inherited mitochondrial disorder, is a multi-organ disease with broad manifestations of stroke-like episodes, epilepsy, lactic acidemia, and myopathy. It is crucial to consider the possibility of mitochondrial disorder, early cardiac screening, and follow-up in young patients with hypertension, hearing loss, and kidney impairment.

## Supplementary Material

ytae690_Supplementary_Data

## Data Availability

The data underlying this article are available in the article and its online Supplementary material.

## Lead author biography

Dr. Novi is a heart failure cardiologist from Indonesia. After graduating from the National Cardiovascular Centre Harapan Kita Jakarta-Faculty of Medicine, University of Indonesia, she honed her skills with a fellowship at the National University Hospital in Singapore. She is currently chairing the heart failure and cardio-oncology programme in South Sumatra Indonesia. She is also an active member of several organizations, such as the Fellow of the American College of Cardiology, Fellow of the Heart Failure Association of the ESC, and Emerging Leader of the Asia Pacific Society of Cardiology. Her research focus includes acute heart failure, HCM, and cardiac amyloidosis.
